# Validation of a new method for the endoscopic measurement of post-bariatric gastric outlet using a standard guidewire: an observer agreement study

**DOI:** 10.1186/s13104-016-2350-6

**Published:** 2017-01-03

**Authors:** Luiz Gustavo de Quadros, Manoel dos Passos Galvão Neto, Josemberg Marins Campos, Roberto Luiz Kaiser Junior, Eduardo Grecco, Mario Flamini Junior, Marcelo Falcao de Santana, Idiberto Jose Zotarelli Filho, Adriano Augusto Tomas Vasconcelos Almeida Alexandre

**Affiliations:** 1 Department of Endoscopy and Bariatric Surgery, Kaiser Clinic and Day Hospital, São José do Rio Preto, SP 15015-110 Brazil; 2 Department of Digestive Surgery, School of Medicine of ABC, Santo Andre, SP 09080-650 Brazil; 3 Brazilian Bariatric Endoscopy International Group, São Paulo, Brazil; 4 Gastro Obeso Center, São Paulo, SP 01308-000 Brazil; 5 Department of Surgery, Federal University of Pernambuco (UFPE), Recife, PE 50670-901 Brazil; 6 State University of Sao Paulo-Unesp-Ibilce, Rua Cristovão Colombo 2265, Sao Jose do Rio Preto, SP 15054-000 Brazil

**Keywords:** Gastric outlet, Observer agreement, Measurement reliability, Weight regain, Weight recidivism, Upper endoscopy

## Abstract

**Background and aims:**

Between 10 and 20% of all patients undergoing bariatric surgery procedures regain weight secondary to a gastrojejunostomy enlargement. The aim of this study was to validate the interobserver agreement while measuring gastric outlet diameters using a new standard guidewire.

**Methods:**

We selected thirty-five videos of consecutive endoscopic procedures on patients undergoing esophagogastroduodenoscopy after a Roux-en-Y gastric bypass procedure. All videos were evaluated by four raters: two expert endoscopists and two trainees. We excluded videos having a slipped Fobi ring or a strictured gastric outlet. Anastomosis diameter was measured using a novel device with standardized markings on a guidewire (Hydra jagwire, Boston Scientific, Natick. MA) as well as the current gold standard defined as a calibrated endoscopic measuring instrument (Olympus America, Center Valley, PA).

**Results:**

We obtained 272 measurements of the gastric outlet. Overall agreement measured through intra-class correlation coefficients for the gold standard was 0.84 (p < 0.01) and 0.83 (p < 0.01) for the new guidewire. Agreement among experts was 0.699 (p < 0.01), while among trainees it was 0.822 (p < 0.01).

**Conclusion:**

The new guidewire demonstrated a high degree of observer reliability, also presenting similar results between expert endoscopists and trainees.

## Background

Although bariatric surgery procedures effectively treat morbid obesity and reduce rates of long-term obesity-related complications [1, 2], weight regain might occur in up to 20% of all patients. Among a multitude of possible factors associated with weight regain, gastric outlet dilation occurs when its diameter exceeds 14–20 mm, ultimately leading patients back to obesity [3]. Although outlet measurement is an essential step in defining a therapeutic plan, to our knowledge there are no previous studies evaluating the reliability associated with different measurement alternatives.

A number of therapeutic options have been developed to reduce the anastomotic diameter, including endoscopic suturing devices and gastrojejunostomy [4–6]. Abnormal anatomical findings are found in 71.2% of all patients, 58.9% of which have a dilated anastomosis, 28.8% present a dilated pouch, and 12.3% with both conditions [7].

Currently, there seems to be no consensus on how to best measure anastomosis diameter. Devices designed to measure these dimensions frequently differ in both units and mechanisms, including balloons, clamps and spacers. While most authors use gaging through a grasper-type forceps connected to the endoscope working channel, [8, 9] no consensus exists on which differences might exist when different measurement tools might be used. In addition, grasper-type forceps are both expensive and hard to find in developing countries.

The objective of this study was therefore to validate a novel, efficient and inexpensive method to measure gastric outlet diameter, evaluating its agreement reliability.

## Methods

### Ethics

This prospective study was approved by the Institutional Review Board of the Hospital Beneficiencia of São José do Rio Preto. Informed consent was obtained by all participants prior to the implementation of any study protocol.

### Cases and measurement

All patients were consecutively recruited through the Digestive Endoscopy Service of the Kaiser Clinic, Brazil. Patients were included if they had undergone gastric bypass surgery with or without a Fobi band. The endoscopic procedure was conducted as a routine exam which is part of our postoperative protocol and regardless of existing symptoms. Patients were excluded if they had been found to have a gastrojejunostomy stenosis or a slipped ring.

After sedation, an upper endoscopic procedure was conducted by an expert endoscopist using an Olympus CV-180 or 160 endoscope (Olympus, Tokyo, Japan). All measurements were recorded in high-definition. All patient identifiers were stripped from the video in order to ensure patient confidentiality.

**Fig. 1:**
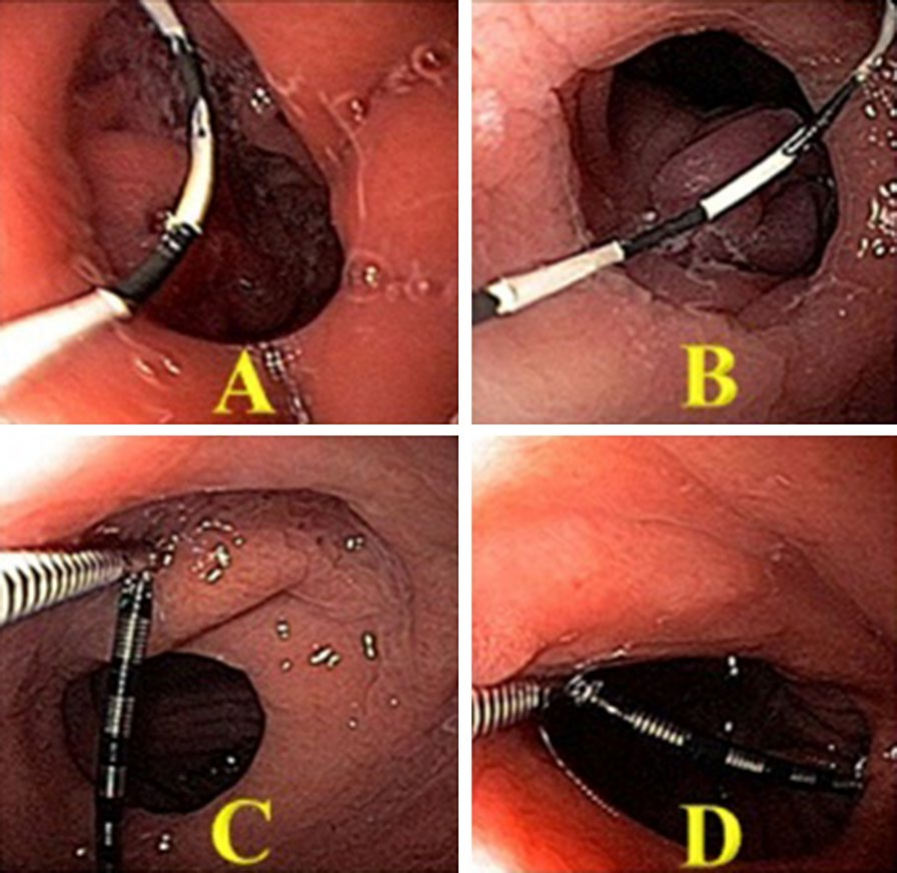
Diameter measurement of the gastrojejunal anastomosis through the guidewire (**A**, **B**) and gold standard method (**C**, **D**)

The anastomosis diameter was initially measured through an articulated device for anastomosis scouting with distal markings at every 0.2 mm by 0.2 mm (Fig. 1).

A second measurement was then conducted with the experimental guidewire, custom manufactured from a Wire Guide Hydra Jagwire (Boston Scientific) with a hydrophilic flexible tip painted in black and additional black stripes every 0.5 mm.

A total of 35 videos from endoscopic exams conducted on 35 patients were rated by four raters, two expert endoscopists and two clinicians in training.

### Data analysis

We started the analysis with a graphical exploration of the sample data, evaluating means and standard deviation for numeric variables, as well as frequencies and percentages for categorical variables. Numeric variables were also evaluated for their distribution and extreme values. The association across ratings was further evaluated through a correlation graphic. Intra-class correlation coefficients were used to evaluate the inter-rater reliability of ratings between novice and expert raters, standard and guidewire, and combinations among the previous categories. Finally, a Bland–Altman plot was used to assess the agreement reliability comparing experts and novice raters.

## Results

No injuries or complications occurred during any of the measurements. Patients’ average age was 38.46 ± 10.61, with 77.14% (n = 27) of them being women.

**Fig. 2:**
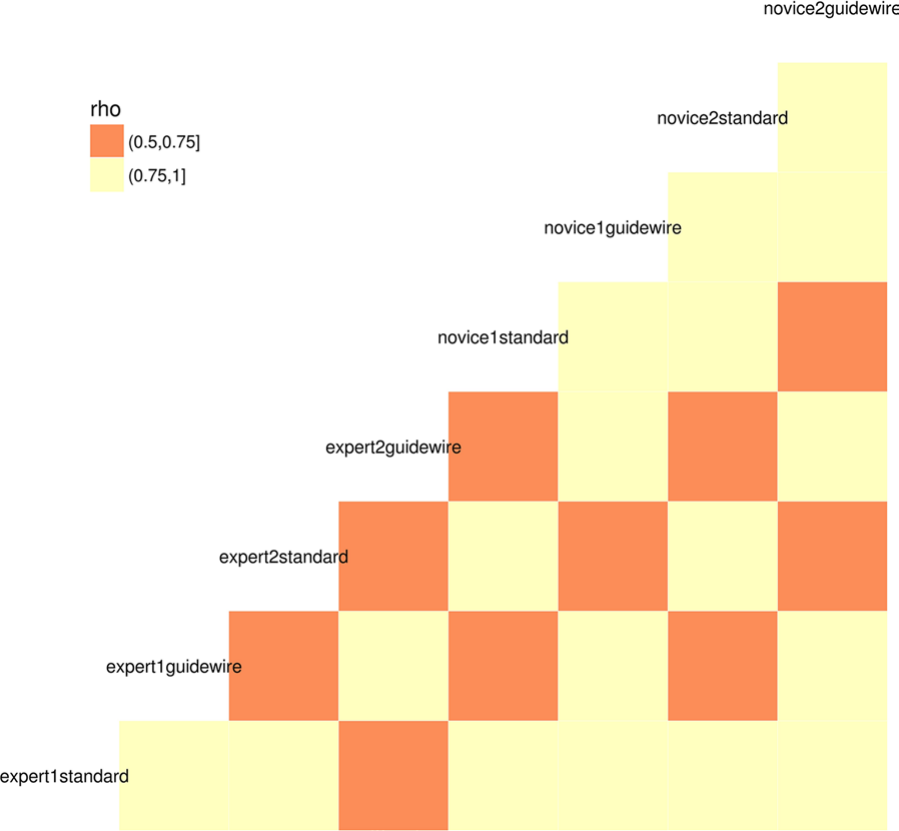
Correlation plot for measurements across raters

**Fig. 3:**
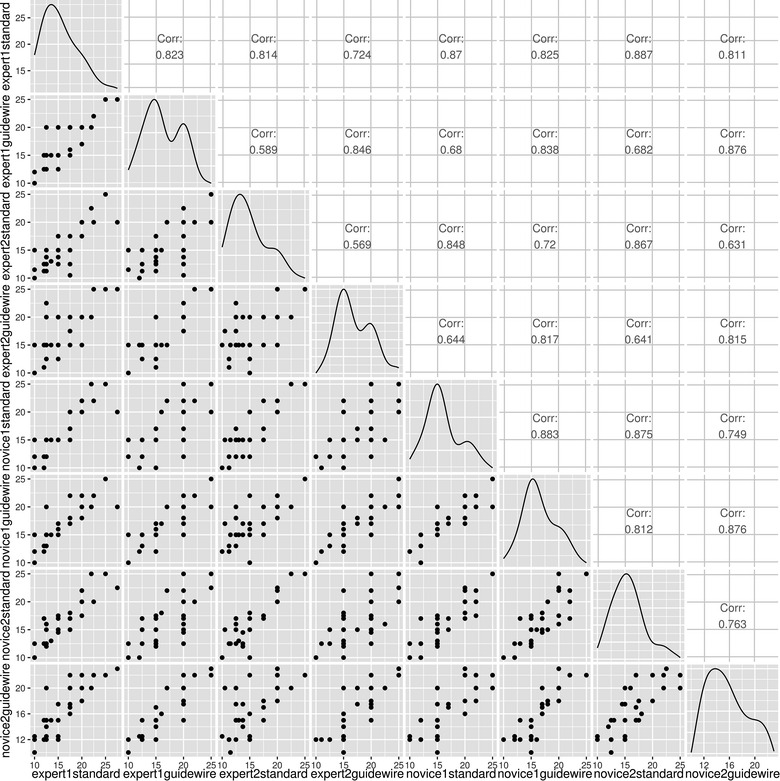
Association among rater measurements

When visually inspecting the correlation among different raters, there was a higher correlation in ratings within experts as well as within novice raters. In addition, the agreement using the guidewire and the gold standard made by the same rater were higher than across other raters (Figs. 2, 3).

**Fig. 4:**
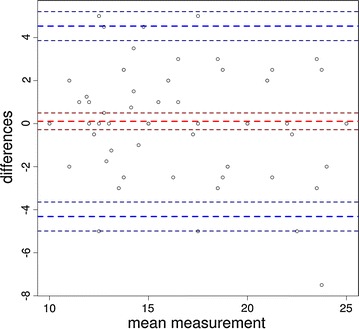
Bland–Altman plot comparing expert and novice raters

Overall agreement measured through intra-class correlation coefficients for the gold standard was 0.84 (p < 0.01) and 0.83 (p < 0.01) for the new guidewire. Agreement among experts was 0.699 (p < 0.01), while among trainees it was 0.822 (p < 0.01). When evaluating subgroups, intra-class correlation coefficients for trainees evaluating the gold standard was 0.877 (p < 0.01), trainees evaluating the new guidewire was 0.865 (p < 0.01), experts evaluating the gold standard was 0.795 (p < 0.01), and experts evaluating the guidewire was 0.843 (p < 0.001). These high rates of agreement are confirmed through the Bland–Altman plot comparing novice and expert raters (Fig. 4). This figure displays the rates of agreement among observers falling within an acceptable range (within the two horizontal lines). The horizontal lines represent the mean difference in agreement plus or minus 1.96 times the standard deviation of the differences.

## Discussion

We found that our novel guidewire instrument has a high agreement reliability in comparison with the gold standard Olympus calibration device. This high agreement reliability is maintained for both expert and novice raters. In addition, no patients demonstrated any complications during the measurement procedures in this sample. These results are important in that the gold standard Olympus device is not only expensive, but of difficult acquisition in developing countries. Our novel device should therefore increase access to this measurement procedure.

In an attempt to avoid weight regain, reducing the diameter of a dilated anastomosis may lead to a reduction of up to 23% in weight [10]. Most studies will recommend a 20 mm diameter, although diameters as small as 12 mm have been proposed [11]. Despite this growing body of evidence regarding the importance of the anastomosis diameter, the literature can be challenged if the measurement process has not been properly validated.

Our results are clinically relevant to clinical practice since approximately 20–30% of all bariatric patients regain weight, many of these being addressed through an endoscopic procedure [12, 13]. The precision in these procedures is essential, in that an anastomosis with a diameter smaller than 5 mm would prevent patients from digesting liquids, a diameter smaller than 10 mm would prevent patients from digesting solid food, while diameters greater than 14 mm are associated with weight regain [11, 14]. The margin for error is therefore small, thus requiring not only measurement precision but also a high degree of reliability across measurements. In addition, it is important for clinicians to communicate with patients so that they can be informed about the current status of their procedure, but also participate in decisions that will affect their ability to eat and drink [15, 16]. In addition, this novel device increases access to this measurement method in countries where the traditional device is either difficult to find or expensive to incorporate into clinical practice.

Although we propose a new and inexpensive and reliable method for measuring dilation, our study does have limitations. First, we evaluated the reliability of videos rather than conducted new endoscopic procedures. While conducting procedures on the same patient by different endoscopists would enhance our methodology, this methodology would pose risks and discomfort to our patients, and was therefore avoided. Second, our sample was small and restricted to a single center. Future studies should therefore further validate our results with more representative samples.

In summary, our study presents a novel, simple, safe, accurate and inexpensive method to measure the outlet diameter. These findings are relevant in that the gold standard Olympus calibration device is not only of difficult access in a number of countries, but also expensive. Given that we have demonstrated equivalent measurement reliability between the Olympus device and our new guidewire, both with experienced endoscopists and trainees, we recommend its use in clinical and research practice.
